# Identification of Candidate Genes for the Plateau Adaptation of a Tibetan Amphipod, *Gammarus lacustris*, Through Integration of Genome and Transcriptome Sequencing

**DOI:** 10.3389/fgene.2019.00053

**Published:** 2019-02-11

**Authors:** Shubo Jin, Chao Bian, Sufei Jiang, Shengming Sun, Lei Xu, Yiwei Xiong, Hui Qiao, Wenyi Zhang, Xinxin You, Jia Li, Yongsheng Gong, Bo Ma, Qiong Shi, Hongtuo Fu

**Affiliations:** ^1^Key Laboratory of Freshwater Fisheries and Germplasm Resources Utilization, Ministry of Agriculture, Freshwater Fisheries Research Center, Chinese Academy of Fishery Sciences, Wuxi, China; ^2^BGI Research Center for Aquatic Genomics, Chinese Academy of Fishery Sciences, Shenzhen, China; ^3^Shenzhen Key Lab of Marine Genomics, Guangdong Provincial Key Lab of Molecular Breeding in Marine Economic Animals, BGI, Shenzhen, China; ^4^Centre of Reproduction, Development and Aging, Faculty of Health Sciences, University of Macau, Taipa, China; ^5^Wuxi Fisheries College, Nanjing Agricultural University, Wuxi, China; ^6^Heilongjiang River Fisheries Research Institute, Chinese Academy of Fishery Sciences, Haebin, China

**Keywords:** *Gammarus lacustris*, *Gammarus pisinnus*, whole-genome sequencing, full-length transcriptome, comparative transcriptome, plateau adaptation

## Abstract

The amphipod *Gammarus lacustris* has been distributing in the Tibetan region with well-known uplifts of the Tibetan plateau. It is hence considered as a good model for investigating stress adaptations of the plateau. Here, we sequenced the whole-genome and full-length transcriptome of *G*. *lacustris*, and compared the transcriptome results with its counterpart *Gammarus pisinnus* from a nearby plain. Our main goal was to provide a genomic resource for investigation of genetic mechanisms, by which *G*. *lacustris* adapted to living on the plateau. The final draft genome assembly of *G*. *lacustris* was 5.07 gigabases (Gb), and it contained 443,304 scaffolds (>2 kb) with an N50 of 2,578 bp. A total of 8,858 unigenes were predicted in the full-length transcriptome of *G*. *lacustris*, with an average gene length of 1,811 bp. Compared with the *G*. *pisinnus* transcriptome, 2,672 differentially expressed genes (DEGs) were up-regulated and 2,881 DEGs were down-regulated in the *G*. *lacustris* transcriptome. Along with these critical DEGs, several enriched metabolic pathways, such as oxidative phosphorylation, ribosome, cell energy homeostasis, glycolysis and gluconeogenesis, were predicted to play essential roles in the plateau adaptation. In summary, the present study provides a genomic basis for understanding the plateau adaption of *G*. *lacustris*, which lays a fundamental basis for further biological and ecological studies on other resident aquatic species in the Tibetan plateau.

## Introduction

Gammaridean amphipods are distributed in almost all aquatic environments, from fresh water to estuarine areas to the deep sea (Lincoln, 1979). As an important component of aquatic ecosystems (Duffy and Hay, 2000), they often act as a model organism for studies on ecotoxicology (Chaumot et al., 2015) and developmental biology (Wolff and Gerberding, 2015). The evolutionary history and current disjunctive distribution of gammarids are largely affected by certain past geological events, such as plate tectonic movements and Tethyan events (Hou et al., 2011, 2014a), suggesting that environmental changes have played an important role in the diversification of gammarid lineages and may have promoted adaptive radiation into new habitats.

Many aquatic species have low survival abilities in serious conditions, such as environments with low or high temperatures, strong light exposure, or low oxygen content. The low survival populations dramatically hinder sustainable development of these aquaculture species. Thus, how aquatic organisms adapt to the serious conditions have received more attention in recent years. For example, some studies have focused on distribution of organisms across pressure gradients to better understand the mechanisms by which they resist the adverse effects of severe pressure.

**Figure 1:**
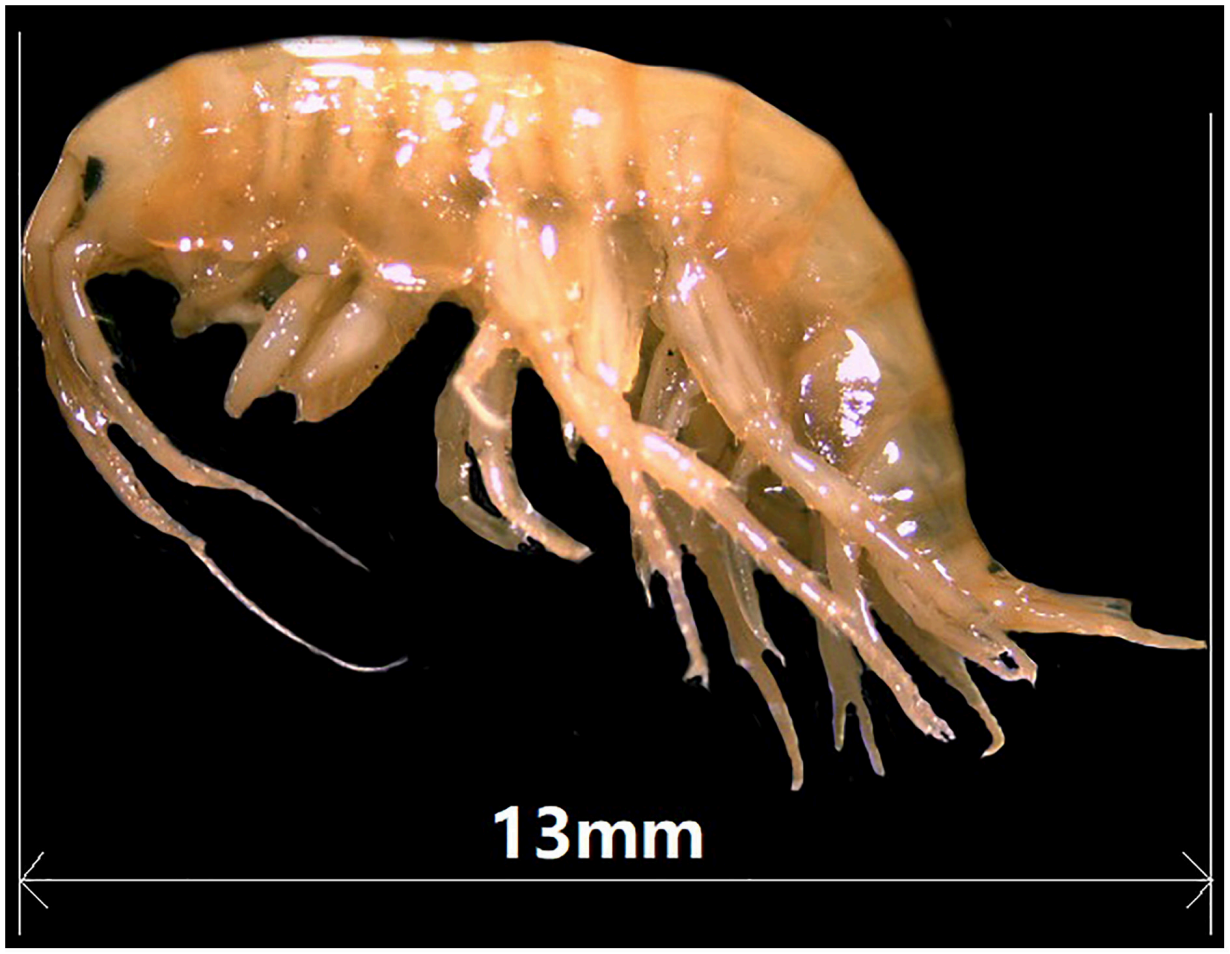
Picture of a *G*. *lacustris*.

Although most Gammaridean amphipod species are highly endemic to a given landmass, *Gammarus lacustris* (Figure 1) is an intercontinental species with a wide distribution in North-western Europe, Russia, North America (Wilhelm and Schindler, 2001; Zadereev et al., 2010), and North-western part of China (Hou et al., 2014b). However, it was also collected by us (in this study) in the Tibet region at an altitude of ~4,300 m, where belongs to the uplifted area of Tibetan plateau. The Tibet region is characterized by serious environmental conditions, including high altitude, low oxygen content, low temperature, and exposure to strong sunlight. Thus, *G*. *lacustris* from Tibet is an excellent model for investigating stress adaptations of plateau. To date, the majority of studies of Tibetan plateau adaptation have been focusing on plants (Jiang et al., 2012; Liu et al., 2014; Li and Du, 2015), and only few animal studies have been conducted. Wang et al. (2014) employed a whole-genome sequencing approach to study the adaptation to hypoxia in dogs and humans on the Tibetan plateau, and they reported couple of candidate genes. In another study, Qu et al. (2013) compared avian genomes to identify genes related to energy metabolism and the immune system that may be involved in the plateau adaptation of the Tibetan ground-tit, *Parus humilis*. Several immune-related studies of *G*. *lacustris* also have been reported. For example, the effects of environmental calcium and flucythrinate on molt cycle and mortality of *G*. *lacustris* were conducted (Richard and Pam, 1984; Rukke, 2002). The effects of parasites (acanthocephalans) on the development of *G*. *lacustris* also have been investigated (Tokeson and Holmes, 1982). However, to the best of our knowledge, no studies have investigated the Tibetan plateau adaptation of *G*. *lacustris* at a genomic level. Although transcriptomic data are available for a few amphipod species (Gismondi and Thomé, 2016; Truebano et al., 2016; Collins et al., 2017), no genome sequences for the genus *Gammarus* have been reported to date.

*G. pisinnus*, collected from Shanxi Province at the altitude of ~510 m, was used for comparative studies in our present work. It has been restricted to the South of the Taihang Mountain, China (Hou et al., 2014b), although both *G. pisinnus* and *G*. *lacustris* are freshwater species (Hou et al., 2014a). Their close relationship and remarkable difference in the residential altitudes make *G. pisinnus* as a reverse counterpart for comparatively analyzing the plateau adaptation of *G*. *lacustris*.

In this study, we performed whole-genome and full-length transcriptome sequencing of *G. lacustris*, and subsequently compared transcriptomes between *G. lacustris* and *G. pisinnus*. The main goal of our project was to provide genetic data to better understand the adaptation of *G. lacustris* to the severe environment of the uplifted Tibetan plateau. These genes and their related metabolic pathways will provide a genetic basis for further biological and ecological studies on *G. lacustris* in the Tibetan plateau.

## Materials and Methods

### Ethics Approval

We obtained permission to collect samples from the Tibet and Shanxi fishery management councils. Neither *G. lacustris* nor *G. pisinnus* is endangered species in China; therefore, both can be used for experimental purposes. All the experimental procedures were approved by the committee of the Freshwater Fisheries Research Center under Chinese Academy of Fishery Sciences.

### Sample Preparation

*Gammarus lacustris* individuals were collected from the Tibetan plateau at the altitude of ~4,300 m (94°48′53″E, 28°41′24″N). To prevent degradation of DNA, entire bodies were stored immediately in 100% ethanol after anesthesia in MS-222, and the ethanol was changed twice before extraction of genomic DNA. For full-length and next-generation transcriptome sequencing, whole individuals were immediately immersed in RNAlater solution (Takara, Tokyo, Japan) after collection, and then were frozen in liquid nitrogen until used for RNA extraction. Meanwhile, *G. pisinnus* individuals for next-generation transcriptome sequencing were collected from a nearby plain in Shanxi Province of China at the altitude of ~510 m (109°7′6″E, 34°5′57″N). Entire shrimps were immediately immersed in RNAlater solution after collection, and then were frozen in liquid nitrogen for storage.

### Genomic DNA Sequencing of *G. lacustris* and *de novo* Genome Assembly

Genomic DNA was extracted from muscle tissue of each *G. lacustris*. Seven paired-end libraries, including three short-insert libraries (250, 500, and 800 bp) and four long-insert libraries (2, 5, 10, and 20 kb), were constructed in accordance with the standard protocol from Illumina (San Diego, CA, USA). An Illumina HiSeq 2000 platform was used for subsequent sequencing of each library. In order to generate a whole-genome assembly, we employed SOAPdenovo2 (v2.04, Luo et al., 2012) with optimized parameters (–K 41) to use the reads from short-insert libraries to construct contigs and original scaffolds. These reads were then aligned for scaffold construction by utilizing the paired-end reads from the long-insert libraries. The reads of short-insert libraries were subsequently used to fill the gaps in the intra-scaffolds.

### Full-Length Transcriptome Sequencing of *G. lacustris* and Transcriptome Annotation

Five *G. lacustris* individuals were pooled to provide sufficient RNA for full-length transcriptome sequencing, with an aim to establish a reference transcriptome for further analysis. UNlQ-10 Column Trizol Total RNA Isolation Kit (Sangon, Shanghai, China) was used to extract total RNA following the manufacturer's instructions. RNA integrity was checked using an Agilent RNA 6000 Nano kit and chips on a Bioanalyzer 2100 (Agilent Technologies, Santa Clara, CA, USA).

Construction of full-length complementary DNA (cDNA) libraries and sequencing on a PacBio RSII platform (Pacific Bioscience Inc., Menlo Park, CA, USA) were performed at the National Instrumentation Center for Environmental Management (NICEM), Seoul National University, Seoul, Korea. In brief, Clontech SMARTer PCR cDNA Synthesis kit (Takara Bio USA Inc., Mountain View, CA, USA) was used to synthesize the first-strand cDNA. Large-scale double-strand cDNA was subsequently amplified with optimal number of polymerase chain reaction (PCR) cycles. Bluepippin™ System (Sage Science Inc., Beverly, MA, USA) was employed to purify and elute the large-scale PCR products to the optimal size (approximately 1~6 kb), and AMPure PB Beads (Pacific Biosciences Inc.) were used to re-purify the large-scale PCR products two more times. SMRTbell library kit (Pacific Biosciences Inc.) was used to prepare the template library. The process, including DNA damage repair, blunt-end ligation, and annealing of the SMRTbell adapters, was performed according to the manufacturer's protocol. The template was eluted to construct libraries of approximately 1~2 kb, 2~3 kb, and 3~6 kb, respectively using the Bluepippin™ system. An Agilent 2100 Bioanalyzer was used to measure the average molecular weight and concentration of each library. All libraries were sequenced in a PacBio RSII platform according to the manufacturer's instructions.

The RS IsoSeq pipeline (version 2.3, Pacific Biosciences, 2015) was used to process the sequence data. Adapters and artifacts were removed to generate reads of inserts (ROIs). Short sequences with length <300 bp were removed. The Bluepippin cDNA size were collected from 500 bp. The ROI sequences were further filtered into two groups, including full-length (FL) ROI sequences and non-FL sequences. The FL ROI sequences were identified based on the presence of a 5′ adapter sequence, a 3′ adapter sequence and poly A tails. The Quiver software module was used to polish the ROI sequences in the isoforms. High-quality (HQ) and low-quality (LQ) isoform sequences were generated through the polishing process of Quiver, and they corresponded to an expected accuracy of >99% or <99%, respectively. LQ outputs (or non-FL coverage sequences) are useful in some cases for correcting errors. After combining the HQ and LQ transcripts, we further processed clustering using CD-HIT-EST (*c* = 0.99) (Fu et al., 2012).

In the subsequent step, we removed the contaminant sequences by stepwise CLC (Cheng et al., 2017a). A number of databases were used for annotation of the LRS isoforms of *G*. *lacustris* (Cheng et al., 2017b). Using Blastp, we aligned the transcriptome factors to the PlnTFDB database (http://plntfdb.bio.uni-potsdam.de/v3.0/), which included 20 plant species with 26,184 sequences. We also mapped the transcriptome factors onto the AnimalTFDB database (http://bioinfo.life.hust.edu.cn/AnimalTFDB/), which included data of 75 animal species. The transcriptome factors were also aligned to the CARD database (https://card.mcmaster.ca/) for selection of immune-resistant related genes. These Blastp results from the above-mentioned steps were filtered using the threshold of E-value ≥ 1e^−10^. Finally, all Blastp results were processed with BLAST2GO (Ashburner et al., 2000) for functional annotation.

### Comparison of *G*. *lacustris* and *G*. *pisinnus* Transcriptomes

We performed comparative transcriptome analysis of *G*. *lacustris* and *G*. *pisinnus* in order to identify the key metabolic pathways and related genes that may be involved in the plateau adaptation of *G*. *lacustris*. Fifteen individuals were collected for both *G*. *lacustris* and *G*. *pisinnus*. That is to say, five individuals were pooled to form one biological replicate, and three biological replicates were sequenced for both *Gammarus* species.

The procedures for extraction of total RNA and the integrity measurement were the same as those described for the full-length transcriptome sequencing. Truseq™ RNA Sample Prep Kit (Illumina) was used to prepare RNA samples for comparative transcriptome analysis. Isolated mRNAs were fragmented into smaller parts using a fragmentation buffer. The first-strand and second-strand cDNAs were synthesized using these short fragments as templates. The short fragments were ligated with sequencing adapters and resolved by agarose gel electrophoresis. Proper fragments were selected and purified for PCR amplification to construct the final cDNA libraries. These cDNA libraries (with insert sizes of approximately 125 bp) for each sample were sequenced from both ends on an Illumina HiSeq 2500 platform.

Clean reads were obtained using the NGS QC TOOLKIT v2.3.3 software (Patel and Jain, 2012). They were assembled into non-redundant transcripts using Trinity program (trinityrnaseq_r20131110, Grabherr et al., 2011). Differences in mRNA levels between *G*. *lacustris* and *G*. *pisinnus* transcriptomes were calculated using RSEM software (Li et al., 2010; Li and Dewey, 2011). Differentially expressed genes (DEGs) were filtered by EB-seq algorithm and subjected to false discovery rate (FDR) analysis with the criteria of FDR < 0.05 (Benjamini et al., 2001).

### Quantitative Real-Time PCR (qRT-PCR) Analysis

Five DEGs predicted to be involved in the plateau adaption of *G*. *lacustris* were further analyzed by qRT-PCR. RNAiso Plus Reagent (Takara, Japan) was used to extract total RNA from both *G*. *lacustris* and *G*. *pisinnus* samples. Concentration and quality of total RNA were measured using a BioPhotometer (Eppendorf, Hamburg, Germany; set qualified A260/A280 values at 1.8~2.0) and 1% agarose gel electrophoresis. Each sample consisted of at least five mature specimens. Experiments were performed in triplicate. iscript™ cDNA Synthesis Kit (Bio-Rad, Hercules, CA, USA) was used to synthesize the first-strand cDNA with approximately 1 μg of total RNA from each sample. Bio-Rad iCycler iQ5 Real-Time PCR System was used to carry out the SYBR Green RT-qPCR assay. Realted primer pairs are listed in Table 1. β*-actin* was used as an internal reference. Diethylpyrocarbonate-treated (DEPC) water for replacement of the cDNA template was used as the negative control. All samples were run in triplicate (each duplicate for target gene and β-actin gene). The 2^−ΔΔCT^ comparative CT method was used to calculate the relative copy number of each gene (Livak and Schmittgen, 2001).

**Table 1:** Primer pairs used for the qRT-PCR validation.

**Primer pairs**	**Sequences**
GADPH-F1	ATTTGAAGGGCGGAGCCAAA
GADPH-R1	CAGGATGCGTTGCTGACAAT
RP1A-F1	TGGATTGCTGTTGCGTTGTG
RP1A-R1	AAGAGGGCACACTTGTCTCG
SC25-F1	GCTCCAAACAGTGTGAACCC
SC25-R1	ATAAACAGCGAAGGCACCCT
Cathepsin-F1	GAGGGTGCCTTCGCTGTTTA
Cathepsin-R1	TTGAAGTAGCCCTTGTCGCC
GST-F1	GAGTACACCGGCACCGTATT
GST-R1	TCCGTCAGCTTTACGTCGTC

*F, forward primer; R, reverse primer*.

One-way analysis of variance was conducted using SPSS software (version 18.0) to analyze the level of significance of any observed differences in gene transcription between *G*. *lacustris* and *G*. *pisinnus*. Statistically significant differences were examined by paired *t*-test, and *P* < 0.05 was considered to be statistically significant.

## Results

### Summary of the *G. lacustris* Genome

The whole-genome sequencing of *G. lacustris* generated 464.41 Gb of raw reads. We ultimately assembled a draft genome of 5.07 Gb, which was composed of 443,304 scaffolds (>2 kb) with an N50 of 2,578 bp; the longest scaffold was 2,165,915 bp. The genome sequencing coverage was estimated to be 91.6 × on the basis of the *K*-mer prediction of 13.5 Gb for the *G. lacustris* genome.

### Full-Length Transcriptome Data for *G. lacustris*

**Figure 2:**
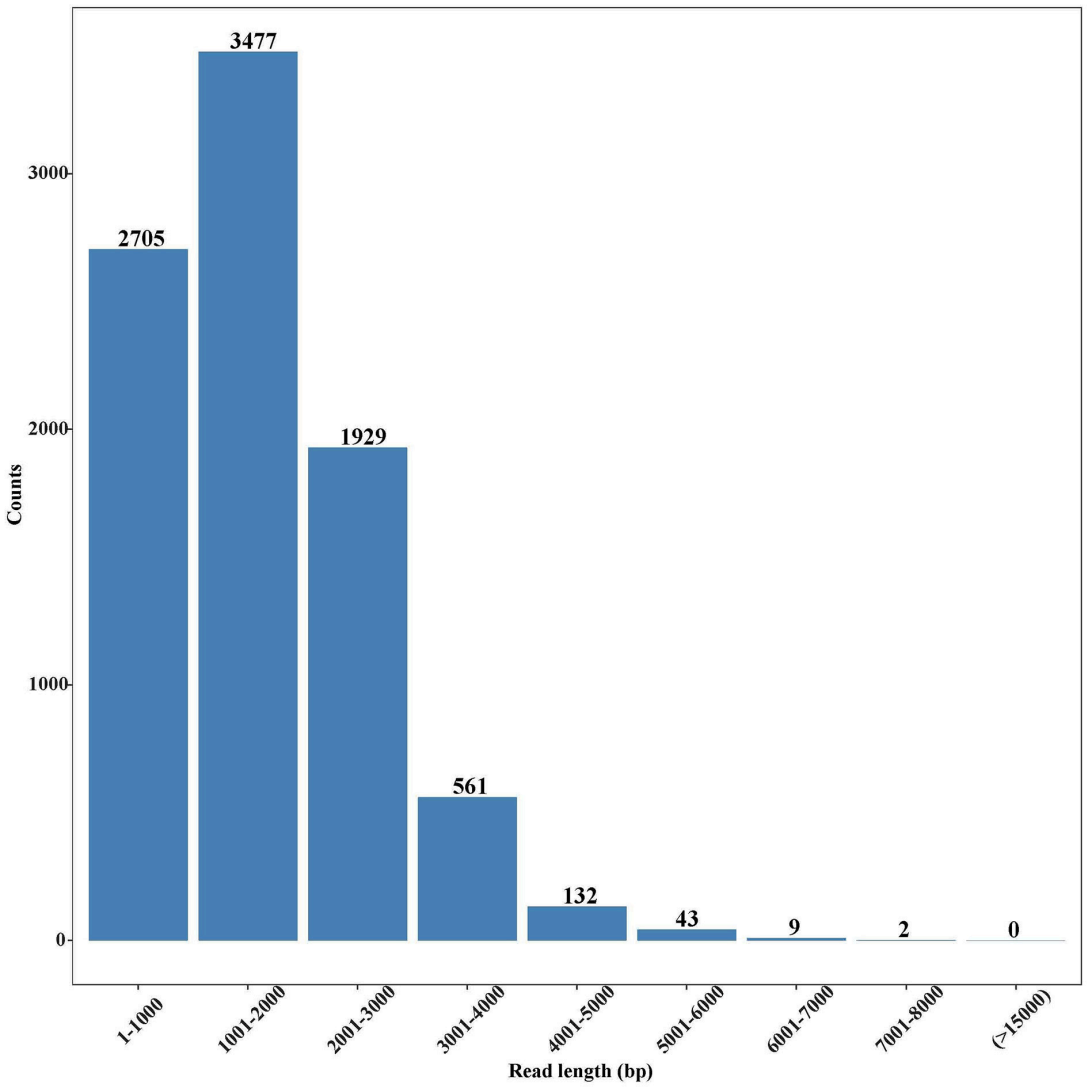
Length distribution of unigenes in the full-length transcriptome of *G*. *lacustris*.

In total, 7,193,977 raw reads were generated, and their sizes ranged from 50 to 74,377 bp with an average of 988 bp. After removal of short sequences (<500 bp), 4,522,837 high-quality reads with the average length of 1,388 bp were retained. These reads were subjected to Blast analysis against the public NCBI non-redundant (nr) database. A total of 8,858 unigenes were identified, with an average length of 1,811 bp (Table S1), and most of them were 1,001–2,000 bp in length (Figure 2). Among these unigenes, 7,166 had coding regions that could be annotated by ESTScan (Iseli et al., 1999) and Blast (Altschul et al., 1997).

### Functional Annotation

**Figure 3:**
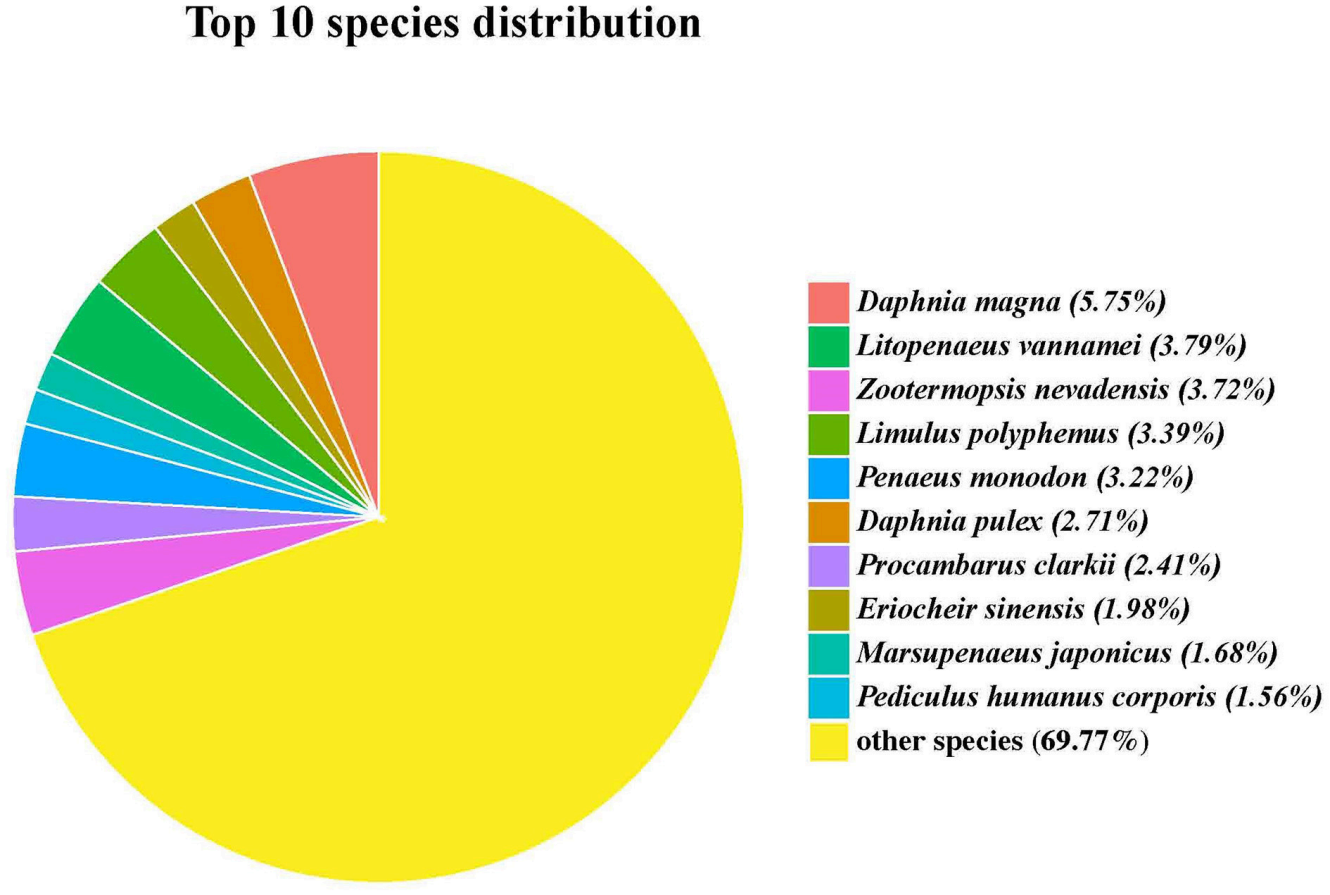
Top 10 hit species for unigenes identified in the full-length transcriptome of *G*. *lacustris*.

Functional annotation of the *G*. *lacustris* unigenes was performed using different databases. A total of 4,008 unigenes (45.25%) were annotated from the NCBI nr database. Most of these unigenes had top hits with the insect and crustacean species. In fact, 229 unigenes matched water flea (*Daphnia magna*), followed by *Litopenaeus vannamei, Zootermopsis nevadensis*, and *Limulus Polyphemus*; the number of unigenes for each species was more than 130 (Figure 3).

**Figure 4:**
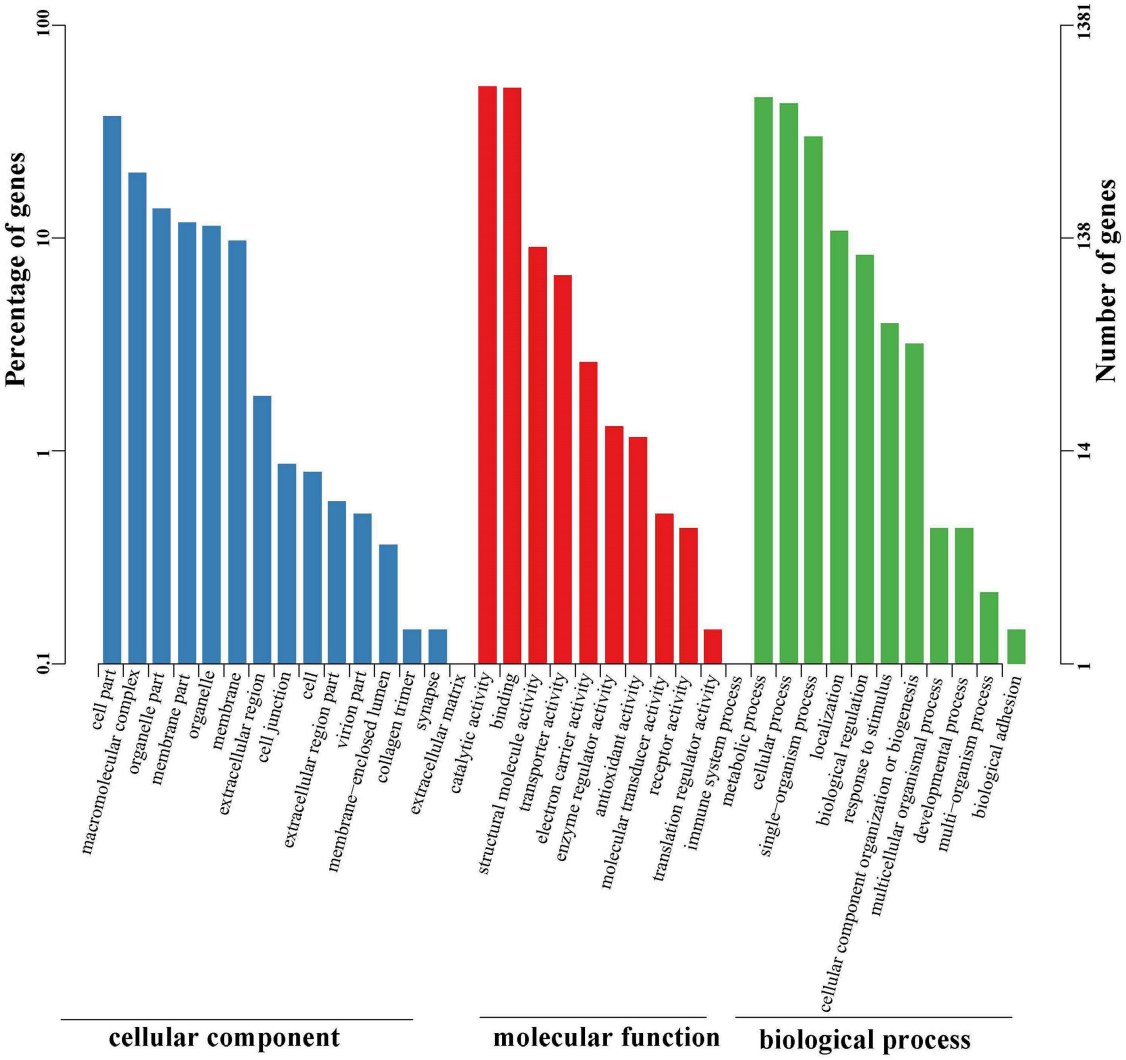
GO classification of unigenes identified in the full-length transcriptome of *G*. *lacustris*. According to the GO terms, we divided 1,381 unigenes into three categories with 37 functional groups, including biological process (12 functional groups), cellular component (15 functional groups), and molecular function (10 functional groups). The left y-axis indicates the percentage of a specific category of genes in the main category, whereas the right y-axis indicates the number of a specific category of genes in the main category.

Functional annotations were further conducted using the public Gene Ontology (GO) and Clusters of Orthologous Groups (COG) databases. These analyses were performed to provide a transparent vocabulary for description of the gene products. A total of 1,381 (15.59%) unigenes were assigned to the GO database (Table S2). Molecular function, cellular component, and biological process were three distinct categories characterized by GO analysis (Figure 4). Molecular function (1,874) was more abundant than biological process (1,235) and cellular component (913). Ultimately, the GO assignments were divided into 37 functional groups, in which catalytic activity (712), binding (700), and metabolic process (633) represented the most abundant.

**Figure 5:**
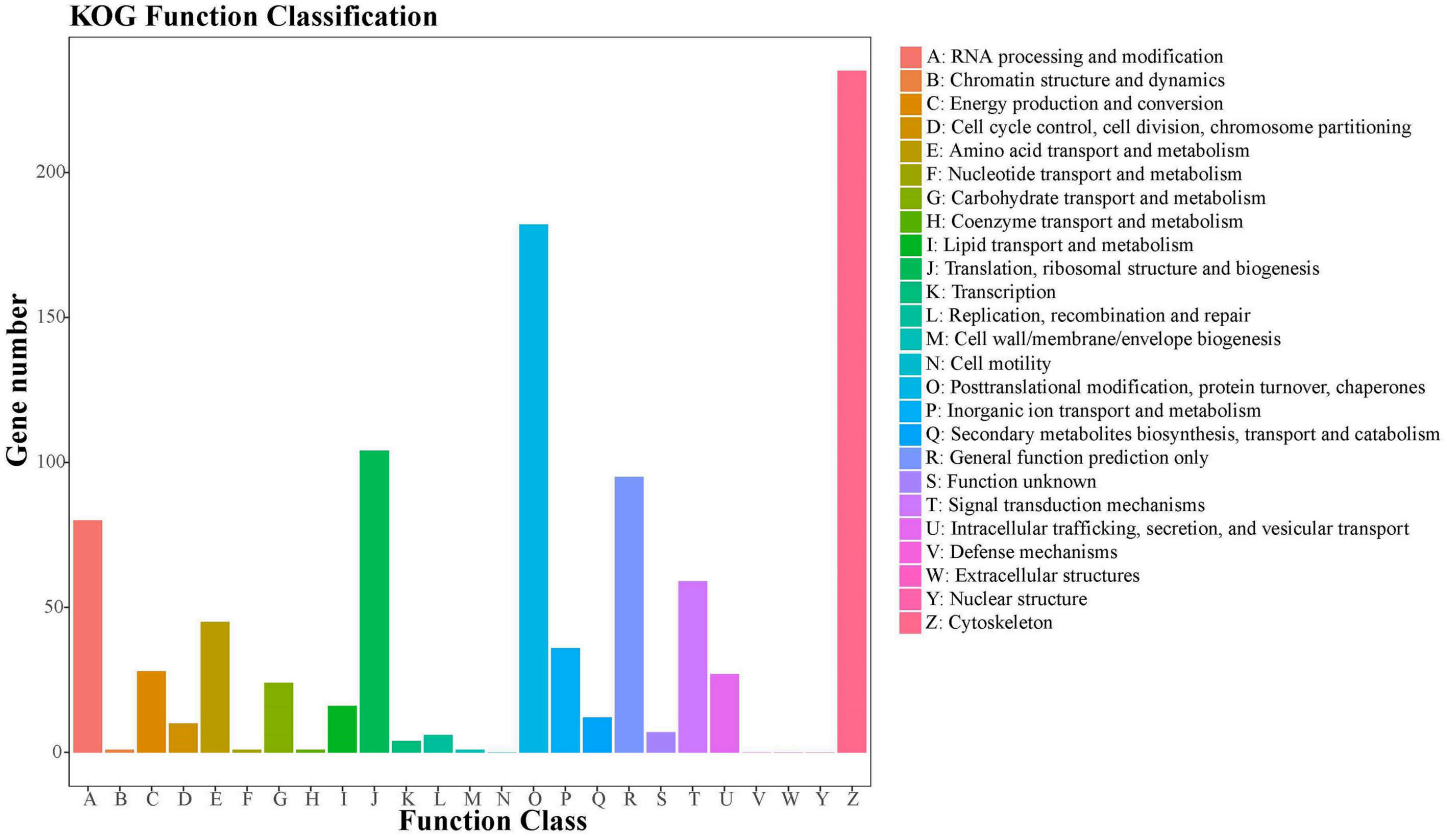
COG classification of putative proteins. A total of 905 putative proteins were classified functionally into 21 molecular families in the COG database.

A total of 905 (10.22%) unigenes were assigned to the COG database, with classification of 21 functional categories (Table S3). Cytoskeleton (235), posttranslational modification, protein turnover, chaperones (182), and translation, ribosomal structure, and biogenesis (104) represented the largest functional categories (Figure 5).

The transcripts involved in biological pathways were predicted and identified using Kyoto Encyclopedia of Genes and Genomes (KEGG) database. In total, 2,422 (27.34%) unigenes were assigned to the KEGG database. These unigenes were further mapped onto 238 predicted metabolic pathways (Table S4), in which oxidative phosphorylation, phagosome, ribosome, and focal adhesion were listed in the top four.

Based on published data from other aquatic species, 13 homolog genes were identified from the full-length transcriptome of *G*. *lacustris* (Table 2) as potentially playing essential roles in maintaining normal life processes of *G*. *lacustris* in the Tibetan region.

**Table 2:** Immune-related unigenes identified in the full-length transcriptome of *G*. *lacustris*.

**Transcriptome**	***E*-value**	**Accession number**	**Species**
AMP-activated protein kinase α	1.60E-171	gb|AKE50479.1|	*Litopenaeus vannamei*
AMP-activated protein kinase β	4.70E-58	gb|AKE50480.1|	*Litopenaeus vannamei*
Glutathione S-transferase	8.80E-61	emb|SAY41336.1|	*Gammarus pulex*
Catalase	5.00E-223	emb|SAA00029.1|	*Gammarus pulex*
serine proteinase inhibitor	4.20E-21	gb|AJR22372.1|	*Eriocheir sinensis*
L-lactate dehydrogenase	1.10E-115	gb|AIM43585.1|	*Halocaridina rubra*
superoxide dismutase copper/zinc	1.50E-44	dbj|BAP28201.1|	*Marsupenaeus japonicus*
Ubiquitin-conjugating enzyme E2	4.50E-91	gb|ACJ03792.1|	*Scylla paramamosain*
E3 Ubiquitin-protein ligase	1.30E-10	ref|XP_004713113.1|	*Echinops telfairi*
Ubiquitin carboxyl-terminal esterase L3	5.10E-43	gb|ALM09354.1|	*Penaeus monodon*
Ubiquitin b	3.00E-61	gb|ADO32980.1|	*Eriocheir sinensis*
60S ribosome subunit	4.70E-65	ref|XP_013399191.1|	*Lingula anatina*
Ribosome like protein	1.80E-73	dbj|BAB78484.1|	*Marsupenaeus japonicus*

### Identification of DEGs From *G*. *lacustris* and *G*. *pisinnus*

All assembled conspecific transcriptomes were clustered into a single file in order to eliminate the effect of redundancy. The final merged data contained 66,796 and 47,531 unigenes for the *G*. *lacustris* and *G*. *pisinnus* transcriptomes, with mean lengths of 824.85 bp and 1,328.45 bp, respectively. However, only 22,688 and 20,130 unigenes were annotated, respectively.

Gene transcription levels were quantified by values of reads per kilobase transcriptome per million mapped reads (RPKM) and compared between *G*. *lacustris* and *G*. *pisinnus*. Ultimately, 5,553 DEGs were identified in the *G*. *lacustris* transcriptome, including 2,672 up-regulated and 2,881 down-regulated genes (Table S5).

A total of 3,178 and 2,793 DEGs were further assigned to GO and KEGG databases, respectively. GO assignments contained 51 different functional groups, in which metabolic process, cellular process, and cell represented the largest. KEGG analysis revealed 291 metabolic pathways, with glycolysis/gluconeogenesis, hypoxia-inducible factor−1 (HIF-1) signaling pathway, RNA degradation, biosynthesis of amino acids, and carbon metabolism as the top pathways (Table S6).

**Figure 6:**
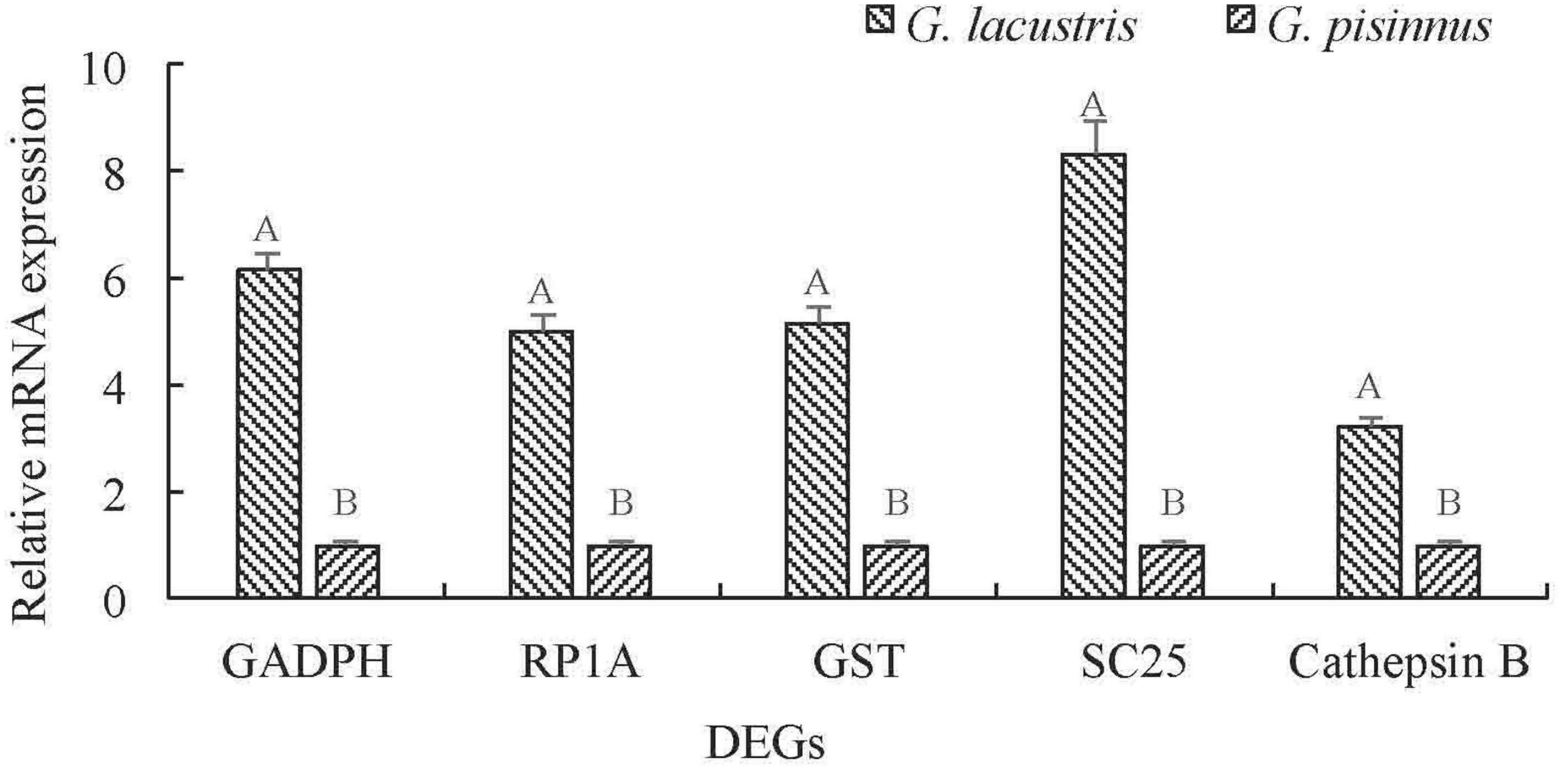
Validation of the relative mRNA transcription of representative DEGs by qRT-PCR. Data are shown as mean ± SD (standard deviation) of tissues from three separate individuals. Capital letters on the bars indicate significant transcriptional differences between the two *Gammarus* species.

We identified several vital DEGs that may be potential candidates for the plateau adaption of *G*. *lacustris* (Table 3). Most of these functional genes were selected out on the basis of comparisons with published data for other aquatic species. However, some were identified based on their dramatic transcriptional difference between *G*. *lacustris* and *G*. *pisinnus*. Five up-regulated DEGs were randomly selected to verify their transcriptional differences by qRT-PCR. The transcription patterns exhibited by these genes from the two *Gammarus* species (Figure 6) are obviously consistent with the RPKM discrepancies obtained from the transcriptome sequencing.

**Table 3:** Strong candidate DEGs for the plateau adaption of *G*. *lacustris*.

**Transcripts**	***E*-value**	**Accession number**	**Fold change (*G. lacustris*/*G. pisinnus*)**	**KEGG pathway**	**Homolog species**
Glyceraldehyde-3-phosphate dehydrogenase (GADPH)	3.00E-181	emb|CAQ60115.1|	10.24324	HIF-1; Carbon metabolism; Glycolysis/Gluconeogenesis; Biosynthesis of amino acids.	*Gammarus locusta*
B-Enolase	2.10E-191	gb|AAF71925.2|	6.48762	HIF-1; RNA degradation; Biosynthesis of amino acids; Carbon metabolism.	*Oryctolagus cuniculus*
Enolase	1.30E-82	ref|XP_018905290.1|	0.32786	HIF-1; RNA degradation; Glycolysis/Gluconeogenesis; Biosynthesis of amino acids; Carbon metabolism.	*Bemisia tabaci*
Glyceraldehyde-3-phosphate dehydrogenase	3.50E-140	ref|XP_005925316.1|	0.293738	HIF-1; Carbon metabolism; Glycolysis/Gluconeogenesis; Biosynthesis of amino acids.	*Haplochromis burtoni*
β-enolase	8.70E-42	ref|XP_016115877.1|	0.26389	HIF-1; Methane metabolism; Glycolysis/Gluconeogenesis; RNA degradation; Biosynthesis of amino acids; Carbon metabolism.	*Sinocyclocheilus grahami*
RING-box protein 1A (RP1A)	8.90E-55	ref|XP_011568300.1|	3.210059	HIF-1; Cell cycle; Ubiquitin mediated proteolysis; Wnt signaling pathway.	*Plutella xylostella*
Solute carrier family 25 (SC25)	1.40E-28	gb|AAH59462.1|	19.94285	Parkinson's disease; Huntington's disease; HTLV-I infection; Calcium signaling pathway.	*Danio rerio*
Cathepsin B-like	1.30E-147	ref|XP_018016997.1|	4.182975	Lysosome; Apoptosis; Renin secretion.	*Hyalella azteca*
5'-AMP-activated protein kinase subunit beta-1-like	1.3E-141	ref|XP_016341003.1|	0.188273	FoxO signaling pathway; Insulin signaling pathway; AMPK signaling pathway; Oxytocin signaling pathway.	*Sinocyclocheilus anshuiensis*
glutathione S-transferase (GST)	3.00E-127	gb|ABV24478.1|	2.187071	Arachidonic acid metabolism	*Hypophthalmichthys molitrix*
Cu/Zn superoxide dismutase	8.60E-82	gb|ADJ67808.1|	0.028581	Prion diseases; Huntington's disease; Peroxisome.	*Hypophthalmichthys molitrix*
Ubiquitin-protein ligase E3B-like	0	ref|XP_016297254.1|	0.043255	Ubiquitin mediated proteolysis.	*Sinocyclocheilus anshuiensis*
Ubiquitin-conjugating enzyme E2 L3-like	1.20E-48	ref|XP_019711983.1|	0.0625	Ubiquitin mediated proteolysis; Parkinson's disease.	*Hippocampus comes*

## Discussion

To the best of our knowledge, this is the first report of whole-genome and full-length transcriptome sequencing of amphipod species. In the present study, we integrated the genome and transcriptome data to support the biological and ecological importance of adaptation of *G*. *lacustris* to the serious environment of the Tibetan plateau. The higher transcription levels of certain genes in *G. lacustris* from the Tibetan plateau compared to those in *G. pisinnus* from a nearby plain suggested that these genes may be potentially involved in the regulatory mechanisms by which *G. lacustris* adapted to living on the plateau.

In this study, the draft genome of *G. lacustris* was 5.07 Gb, which covered 37.55% of the estimated genome and included 443,304 scaffolds (>2 kb) with an N50 of 2,578 bp. Whole-genome sequencing was performed previously in many aquatic species. For example, the assembled genome of common carp (*Cyprinus carpio*) was 1.69 Gb, which covered at least 92.3% of the estimated genome and had a scaffold N50 size of 1.0 Mb (Xu et al., 2014). The assembled genome of female and male grass carp (*Ctenopharyngodon idellus*) were 0.9 and 1.07 Gb respectively, with a scaffold N50 size of 6.4 Mb (Wang et al., 2015). The final genome of half-smooth tongue sole (*Cynoglossus semilaevis*) was 477 Mb, with a scaffold N50 size of 867 kb (Chen et al., 2014). However, the genomes of crustacean species with higher heterozygosity and more repeat sequences were more complex than most fishes. As we know, only one *crustacean* genome was reported. In our previously work (Song et al., 2016), the assembled genome of Chinese mitten crab (*Eriocheir sinensis*) was 1.12 Gb, which covered about 67.47% of the estimated genome and had a scaffold N50 size of 224 kb. Compared with published genomes of aquatic species, the quality of our present draft genome of *G. lacustris* was relatively low. A reasonable explanation is that the big and complex genome of *G. lacustris* is difficult for the next-generation sequencing. A PacBio genome sequencing was also failed to generate high-quality sequences because of difficulty to extract good genomic DNA from *G. lacustris*. However, as the first shrimp genome for a public reference, our achieved data are still useful for comparative biological and genomic studies of Tibetan animals.

### Identification of the Potentially Important Pathways and Genes for Maintaining Normal Life of *G. lacustris* in the Tibetan Plateau

In total, 8,858 unigenes were identified from the full-length transcriptome of *G*. *lacustris*. Oxidative phosphorylation was the main metabolic pathway annotated in this study. This phenomenon occurs in almost all aerobic organisms (Mitchell and Moyle, 1967; Schägger and Pfeiffer, 2001). Oxidative phosphorylation releases and produces adenosine triphosphate (ATP) by using enzymes to oxidase nutrients (Dimroth et al., 2000), and therefore it is the most efficient way to release energy. *G*. *lacustris* likely requires more energy to maintain normal life processes in the serious environmental conditions of the Tibetan region.

Ribosome was another main pathway annotated in the full-length transcriptome of *G. lacustris*. Ribosomes are complex machines present in all living cells, and they are where biological protein synthesis takes place (Benne and Sloof, 1987). Ribosomes link amino acids together as specified by mRNA molecules. Ribosomes are composed of small 40S and large 60S ribosomal subunits. The small subunits read RNAs, whereas the large subunits join amino acids to form a polypeptide chain.

AMP-activated protein kinase (AMPK) is a critical enzyme with essential roles in cellular energy homeostasis. It is generally distributed in liver, skeletal muscle and brain. The important functions of AMPK activation include stimulation of skeletal muscle fatty acid oxidation and glucose uptake, stimulation of hepatic fatty acid oxidation, ketogenesis, and inhibition of cholesterol/triglyceride synthesis and lipogenesis (Winder and Hardie, 1999). AMPK contains three different subunits (α, β, and γ), and each of them is critical for both stability and activity of AMPK (Stapleton et al., 1996). For example, the sensitivity of hypothalamic neurons and pancreatic β cells decreases if the AMPK α subunit is absent in these cells, leading to changes in extracellular glucose concentration (Claret et al., 2007; Beall et al., 2010, 2012; Sun et al., 2010).

In our current study, several ubiquitin gene families were identified. The ubiquitin-proteasome affects protein homeostasis maintenance and cell degradation. It can promote or prevent protein interactions by marking them for degradation by proteasomes (Glickman and Ciechanover, 2002; Schnell and Hicke, 2003; Mukhopadhyay and Riezman, 2007). E3 ubiquitin-protein ligase (E3) regulates the binding of target protein substrate, while the ubiquitin from the ubiquitin-conjugating enzyme (E2) cysteine subsequently is transferred to a lysine residue on the target protein (Ardley and Robinson, 2005). Shen et al. (2008) reported that the transcription level of E2 transcript differed significantly among various stages of testis and ovary development, thus it plays vital roles in oogenesis and spermatogenesis.

Several important antioxidants were also identified in the full-length transcriptome of *G. lacustris*, such as superoxide dismutase (SOD), glutathione S-transferase (GST) and catalase (CAT). Antioxidants have dramatic effects on survival and normal development. They can protect DNAs, cytomembranes and proteins from damage by reactive oxygen species in living animals (Kong et al., 2011; Xi et al., 2015). SOD removes and eliminates the negative effects of free radicals. Thus, SOD content is a vital index of senility and death (Morris and Albright, 1981; Mila-Kierzenkowska et al., 2005; Vutukuru et al., 2006). CAT accounts for about 40% of all expressed peroxidases. It can be found in every tissue, with especially high expression in the liver. CAT can catalyze H_2_O_2_ to H_2_O and O_2_. Thus, H_2_O_2_ cannot bind with O_2_ to form hydroxyl radicals, which are very harmful products in biological systems (Morris and Albright, 1981; Rudneva, 1999; Vutukuru et al., 2006). GST can conjugate the reduced form of glutathione to xenobiotic substrates for detoxification (Sheehan et al., 2001; Udomsinprasert et al., 2005; Allocati et al., 2009). In summary, these three antioxidants likely have positive effects on the immune system of *G. lacustris*, which would help them maintain normal life processes in the Tibetan plateau.

### Critical Candidate DEGs for the Plateau Adaption of *G*. *lacustris*

Several important metabolic pathways and DEGs were identified by comparing the transcriptomes of *G. lacustris* and *G. pisinnus*. Without doubt, hypoxia is one of the most serious environmental factors in the Tibetan plateau. The HIF-1 signaling pathway was identified as one of the most enriched metabolic pathways in this study. This signaling pathway plays an essential role in hypoxic conditions (Semenza, 2004; Benizri et al., 2008; Formenti et al., 2010). In general, HIFs play vital roles in development and in the regulation of human metabolism (Benizri et al., 2008). In mammals, deletion of HIF-1 genes results in perinatal death (Formenti et al., 2010). Formenti et al. (2010) also reported that HIF-1 is vital to chondrocyte survival, as it can allow cells to survive in low-oxygen conditions within the growth plates of bones.

Glycolysis/gluconeogenesis was another main metabolic pathway that was enriched with DEGs. It is involved in maintenance of the normal blood glucose levels in humans and many other animals (Young, 1977). Glucose (C_6_H_12_O_6_) is generated from non-carbohydrate carbon substrates, and it is converted into pyruvate (CH3COCOO-) via glycolysis (Lubert, 1995). NADH (reduced nicotinamide adenine dinucleotide) and high-energy ATP molecules are subsequently formed with the release of free energy. Therefore, glycolysis/ gluconeogenesis may be important for providing energy for *G*. *lacustris* to survive in the serious environment of the Tibetan plateau.

Several DEGs were involved in these metabolic pathways at the same time. For example, glyceraldehyde 3-phosphate dehydrogenase (GAPDH) generates products that are involved in carbohydrate metabolism, thereby catalyzing a vital energy-yielding step. It occurs through the reversible oxidative phosphorylation of glyceraldehyde-3-phosphate when inorganic phosphate and NAD are present (Rius et al., 2008). Energy and molecular carbon are released when GADPH is catalyzed to break down glucose (Tarze et al., 2007). Thus, we predict that GADPH may be a key gene involved in environmental adaption in *G. lacustris*. Enolase is also known as phosphopyruvate hydratase, and it plays essential roles in the ninth and penultimate step of glycolysis, when 2-phosphoglycerate is converted to phosphoenolpyruvate (Hoorn et al., 1974; Zhang et al., 1997). Its role is to cleave carbon-oxygen bonds. Three subunits have been identified (α, β, and γ) (Peshavaria and Day, 1991; Pancholi, 2001), in which β-enolase is a muscle-specific enolase as indicated by its high level in muscle (Peshavaria and Day, 1991).

Five up-regulated genes were randomly selected to verify their transcriptional differences by qRT-PCR. These genes are up-regulated in *G*. *lacustris* and therefore may be involved in the plateau adaption. Transcription patterns of these five DEGs were confirmed to be the same as those from RNA-Seq, indicating that our RNA-Seq data were reliable. Cathepsin B may be involved in specific immune resistance (Fais, 2007) by playing an important role in intracellular proteolysis (Sloane, 1990). It is up-regulated in many diseases, including various pathological conditions, as well as in premalignant lesions and cancers (Lai et al., 2004; Ha et al., 2010; Tong et al., 2014; Yang et al., 2016). Solute carrier family 25 may be involved in the physiological “proton leak” in the liver. This gene is overexpressed when the mitochondrial membrane is potentially dissipated (Tan et al., 2004). RING-box protein 1 is a component of the SCF E3 ubiquitin ligase complex. It plays essential roles in mitotic chromosomal condensation, meiosis and cytokinesis, and it also mediates the ubiquitination and degradation of target proteins. It recruits the E2 ubiquitination enzyme to the complex through the RING-type zinc finger and brings it into close proximity to the substrate (Noureddine et al., 2002).

## Conclusions

In summary, the present study integrated whole-genome and full-length transcriptome sequencing to reveal candidate genes for potential involvement in the plateau adaptation of *G. lacustris*. Several key metabolic pathways and DEGs were identified by comparing the transcriptomes of *G. lacustris* (a resident of the Tibetan plateau) and *G. pisinnus* (a counterpart from a nearby plain). We also observed that energy-metabolism related metabolic pathways and genes are more likely involved in the plateau adaption. A reasonable explanation for this finding is that more energy is necessary for *G. lacustris* to live in the low-temperature environment of the Tibetan plateau, which is consistent with the results from other Tibetan plateau adaptation studies. Our results of this study also provide large data-based genetic resources for future studies on other aquatic species.

## Data Deposition

The PacBio RSII platform reads of *G*. *lacustris* were submitted to NCBI with the accession number of SRP133982. The Illumina Hiseq 2500 platform reads of *G*. *lacustris* and *G*. *pisinnus* were submitted to NCBI with the accession number of SRP132243 and SRP132319, respectively.

## Author Contributions

HF and QS designed the project. LX, YX, HQ, WZ, YG, and BM collected the samples and participated in figure preparation. CB, XY, and JL performed data analysis. ShJ, CB, SuJ, and SS prepared the manuscript. QS and HF revised the manuscript.

### Conflict of Interest Statement

The authors declare that the research was conducted in the absence of any commercial or financial relationships that could be construed as a potential conflict of interest.

## Supplementary Material

Table S1 Summary of BLASTx results for unigenes of the full-length transcritpome of G. lacustris.

Table S2 Categories of Gene Ontology of *G. lacustris* full-length unigenes.

Table S3 Categories of COG of *G. lacustris* full-length unigenes.

Table S4 KEGG summary of *G. lacustris* full-length unigenes.

Table S5 Summary of the differentially expressed genes between *G. lacustris and G*. *pisinnus*.

Table S6 KEGG summary of the differentially expressed genes between *G. lacustris* and *G. pisinnus*.

## References

[B1] Allocati N. Federici L. Masulli M. Di Ilio C. (2009). Glutathione transferases in bacteria. FEBS J. 276, 58–75. 10.1111/j.1742-4658.2008.06743.x 19016852

[B2] Altschul S. F. Madden T. L. Schäffer A. A. Zhang J. Zhang Z. Miller W.. (1997). Gapped BLAST and PSI-BLAST: a new generation of protein database search programs. Nucleic Acids Res. 25, 3389–3402. 10.1093/nar/25.17.3389 9254694 PMC146917

[B3] Ardley H. C. Robinson P. A. (2005). E3 ubiquitin ligases. Essays Biochem. 41, 15–30. 10.1042/bse0410015 16250895

[B4] Ashburner M. Ball C. A. Blake J. A. Botstein D. Butler H. Cherry J. M.. (2000). Gene ontology: tool for the unification of biology. Nat. Genet. 25, 25–29. 10.1038/75556 10802651 PMC3037419

[B5] Beall C. Hamilton D. L. Gallagher J. Logie L. Wright K. Soutar M. P.. (2012). Mouse hypothalamic GT1-7 cells demonstrate AMPK-dependent intrinsic glucose-sensing behaviour. Diabetologia 55, 2432–2444. 10.1007/s00125-012-2617-y 22760787 PMC3411292

[B6] Beall C. Piipari K. Al-Qassab H. Smith M. A. Parker N. Carling D.. (2010). Loss of AMP-activated protein kinase alpha2 subunit in mouse beta-cells impairs glucose-stimulated insulin secretion and inhibits their sensitivity to hypoglycaemia. Biochem. J. 429, 323–333. 10.1042/BJ20100231 20465544 PMC2895783

[B7] Benizri E. Ginouvès A. Berra E. (2008). The magic of the hypoxia-signaling cascade. Cell. Mol. Life Sci. 65(7–8), 1133–1149. 10.1007/s00018-008-7472-0 18202826 PMC11131810

[B8] Benjamini Y. Drai D. Elmer G. Kafkafi N. Golani I. (2001). Controlling the false discovery rate in behavior genetics research. Behav. Brain. Res. 125(1–2), 279–284. 10.1016/S0166-4328(01)00297-2 11682119

[B9] Benne R. Sloof P. (1987). Evolution of the mitochondrial protein synthetic machinery. BioSystems. 21, 51–68. 10.1016/0303-2647(87)90006-2 2446672

[B10] Chaumot A. Geffard O. Armengaud J. Maltby L. (2015). Gammarids as reference species for freshwater monitoring. Aquat. Ecotox. 11, 253–280. 10.1016/B978-0-12-800949-9.00011-5

[B11] Chen S. Zhang G. Shao C. Huang Q. Liu G.. (2014). Whole-genome sequence of a flatfish provides insights into ZW sex chromosome evolution and adaptation to a benthic lifestyle. Nat. Genet. 46, 253–260. 10.1038/ng.2890 24487278

[B12] Cheng B. Furtado A. Henry R. (2017a). Processing of Pacbio Isoseq Sequences. 10.17504/protocols.io.ja3cign

[B13] Cheng B. Furtado A. Henry R. (2017b). Transcriptome Annotation. 10.17504/protocols.io.ja4cigw26226425

[B14] Claret M. Smith M. A. Batterham R. L. Selman C. Choudhury A. I. Fryer L. G.. (2007). AMPK is essential for energy homeostasis regulation and glucose sensing by POMC and AgRP neurons. J. Clin. Invest. 117, 2325–2336. 10.1172/JCI31516 17671657 PMC1934578

[B15] Collins M. Tills O. Spicer J. I. (2017). De novo transcriptome assembly of the amphipod *Gammarus chevreuxi* exposed to chronic hypoxia. Mar. Genom. 33, 17–19. 10.1016/j.margen.2017.01.006

[B16] Dimroth P. Kaim G. Matthey U. (2000). Crucial role of the membrane potential for ATP synthesis by F(1)F(o) ATP synthases. *J. Exp*. Biol. 203(Pt 1), 51–59. 10.1242/jeb.203.1.51 10600673

[B81] Duffy J. E. Hay M.E. (2000). Strong impacts of grazing amphipods on the organization of a benthic community. Ecol. Monogr. 70, 237–263. 10.2307/2657176

[B17] Fais S. (2007). Cannibalism: a way to feed on metastatic tumors. Cancer Lett. 258, 155–164. 10.1016/j.canlet.2007.09.014 17977647

[B18] Formenti F. Constantin-Teodosiu D. Emmanuel Y. Cheeseman J. Dorrington K. L. Edwards L. M.. (2010). Regulation of human metabolism by hypoxia-inducible factor. Proc. Natl. Acad. Sci. U.S.A. 107, 12722–12727. 10.1073/pnas.1002339107 20616028 PMC2906567

[B19] Fu L. Niu B. Zhu Z. Wu S. Li W. (2012). CD-HIT: accelerated for clustering the next-generation sequencing data. Bioinformatics 28, 3150–3152. 10.1093/bioinformatics/bts565 23060610 PMC3516142

[B20] Gismondi E. Thomé J. P. (2016). Transcriptome of the freshwater amphipod *Gammarus pulex* hepatopancreas. Genom. Data 8, 91–92. 10.1016/j.gdata.2016.04.002 27222807 PMC4856825

[B21] Glickman M. H. Ciechanover A. (2002). The ubiquitin-proteasome proteolytic pathway: destruction for the sake of construction. Physiol. Rev. 82, 373–428. 10.1152/physrev.00027.2001 11917093

[B22] Grabherr M. G. Haas B. J. Yassour M. Levin J. Z. Thompson D. A. Amit I. (2011). Trinity: reconstructing a full-length transcriptome without a genome from RNA-Seq data. Nat. Biotechnol. 29, 644–652. 10.1038/nbt.1883 21572440 PMC3571712

[B23] Ha S. D. Ham B. Mogridge J. Saftig P. Lin S. Kim S. O. (2010). Cathepsin B-mediated autophagy flux facilitates the anthrax toxin receptor 2-mediated delivery of anthrax lethal factor into the cytoplasm. J. Biol. Chem. 285, 2120–2129. 10.1074/jbc.M109.065813 19858192 PMC2804368

[B24] Hoorn R. K. Flickweert J. P. Staal G. E. (1974). Purification and properties of enolase of human erythroctyes. Int. J. Biochem. 5(11–12), 845–852. 10.1016/0020-711X(74)90119-0

[B25] Hou Z. Li J. Li S. (2014b). Diversification of low dispersal crustaceans through mountain uplift: a case study of *Gammarus* (Amphipoda: Gammaridae) with descriptions of four novel species. Zool. J. Linn. Soc. 170, 591–633. 10.1111/zoj.12119

[B26] Hou Z. Sket B. Fišer C. Li S. (2011). Eocene habitat shift from saline to freshwater promoted Tethyan amphipod diversification. Proc. Natl. Acad. Sci. USA. 108, 14533–14538. 10.1073/pnas.1104636108 21844362 PMC3167504

[B27] Hou Z. Sket B. Li S. (2014a). Phylogenetic analyses of *Gammaridae crustacean* reveal different diversification patterns among sister lineages in the Tethyan region. Cladistics 30, 352–365. 10.1111/cla.12055 34794244

[B28] Iseli C. Jongeneel C. V. Bucher P. (1999). ESTScan: a program for detecting, evaluating, and reconstructing potential coding regions in EST sequences. Proc. Int. Conf. Intell. Syst. Mol. Biol. 1999, 138–148. 10786296

[B29] Jiang C. M. Yu G. R. Li Y. N. Cao G. M. Yang Z. P. (2012). Nutrient resorption of coexistence species in alpine meadow of the qinghai-tibetan plateau explains plant adaptation to nutrient-poor environment. Ecol. Eng. 44, 1–9. 10.1016/j.ecoleng.2012.04.006

[B30] Kong X. H. Wang S. P. Jiang H. X. Nie G. X. Li X. J. (2011). Changes of the activities of enzymes related to immunity and the content of malondialdehyde during embryonic development of gold-fish *Carassius auratus. J*. *Fish. Sci*. China 18, 1293–1298. 10.3724/SP.J.1118.2011.01293

[B31] Lai W. F. Chang C. H. Tang Y. Bronson R. Tung C. H. (2004). Early diagnosis of osteoarthritis using cathepsin B sensitive near-infrared fluorescent probes. Osteoarthritis. Cartilage 12, 239–244. 10.1016/j.joca.2003.11.005 14972341

[B32] Li B. Dewey C. N. (2011). RSEM: accurate transcript quantification from RNA-Seq data with or without a reference genome. BMC. Bioinformatics 12:323 10.1186/1471-2105-12-323 21816040 PMC3163565

[B33] Li B. Ruotti V. Stewart R. M. Thomson J. A. Dewey C. N. (2010). RNA-Seq gene expression estimation with read mapping uncertainty. Bioinformatics 26, 493–500. 10.1093/bioinformatics/btp692 20022975 PMC2820677

[B34] Li Z. T. Du G. Z. (2015). Ecological sdaptation of the seed microsculptures of *Saussurea* from different altitudes (Qinghai-Tibet Plateau). Pol. J. Ecol. 63, 593–598. 10.3161/15052249PJE2015.63.4.011

[B35] Lincoln R. J. (1979). British Marine Amphipoda: Gammaridea. London: British Museum (Natural History).

[B36] Liu J. Q. Duan Y. W. Hao G. Ge X. J. Sun H. (2014). Evolutionary history and underlying adaptation of alpine plants on the Qinghai-Tibet Plateau. J. Syst. Evol. 52, 241–249. 10.1111/jse.12094

[B37] Livak K. J. Schmittgen T. D. (2001). Analysis of relative gene expression data using realtime quantitative PCR and the 2-ΔΔCT method. Methods 25, 402–428. 10.1006/meth.2001.1262 11846609

[B38] Lubert S. (ed.). (1995). Glycolysis, in Biochemistry, 4th Edn (New York, NY: W.H. Freeman and Company), 483–508.

[B39] Luo R. Liu B. Xie Y. Li Z. Huang W. Yuan J.. (2012). SOAPdenovo2: an empirically improved memory-efficient short-read *de novo* assembler. Gigascience 1:18. 10.1186/2047-217X-1-18 23587118 PMC3626529

[B40] Mila-Kierzenkowska C. Wozniak A. Wozniak B. Drewa G. Chesy B. Drewa T. (2005). Activity of superoxide dismutase (SOD) and concentration of thiobarbituric acid reactive substances (TBARS) in liver and muscles of some fish. *Acta Biol*. Hung. 56(3–4), 399–401. 10.1556/ABiol.56.2005.3-4.21 16196213

[B41] Mitchell P. Moyle J. (1967). Chemiosmotic hypothesis of oxidative phosphorylation. Nature 213, 137–139. 10.1038/213137a0 4291593

[B42] Morris S. M. Albright J. T. (1981). Superoxide dismutase, catalase, and glutathione peroxidase in the swim bladder of the physoclistous fish, Opsanus tau L. Cell Tissue Res. 220, 739–752. 10.1007/BF00210458 7296650

[B43] Mukhopadhyay D. Riezman H. (2007). Proteasome-independent functions of ubiquitin in endocytosis and signaling. Science 315, 201–205. 10.1126/science.1127085 17218518

[B44] Noureddine M. A. Donaldson T. D. Thacker S. A. Duronio R. J. (2002). Drosophila Roc1a encodes a RING-H2 protein with a unique function in processing the Hh signal transducer Ci by the SCF E3 ubiquitin ligase. Dev. Cell 2, 757–770. 10.1016/S1534-5807(02)00164-8 12062088

[B45] Pacific Biosciences (2015). RS IsoSeq (v2.3) Tutorial 2. 2. Isoform level clustering (ICE and Quiver). Available online at: https://github.com/ PacificBiosciences/cDNA primer/wiki/RS IsoSeq-%28v2.3%

[B46] Pancholi V. (2001). Multifunctional alpha-enolase: its role in diseases. Cell. Mol. Life Sci. 58, 902–920. 10.1007/PL00000910 11497239 PMC11337373

[B47] Patel R. K. Jain M. (2012). NGS QC Toolkit: a toolkit for quality control of next generation sequencing data. PLoS ONE 7:e30619. 10.1371/journal.pone.0030619 22312429 PMC3270013

[B48] Peshavaria M. Day I. N. (1991). Molecular structure of the human muscle-specific enolase gene (ENO3). *Biochem*. J. 275(Pt 2), 427–433. 10.1042/bj2750427 PMC1150071 1840492

[B49] Qu Y. Zhao H. Han N. Zhou G. Song G. Gao B. (2013). Ground tit genome reveals avian adaptation to living at high altitudes in the tibetan plateau. Nat. Commun. 4:2071. 10.1038/ncomms3071 23817352

[B50] Richard L. A. Pam S. (1984). Toxicity of flucythrinate to *Gammarus lacustris* (Amphipoda), *Pteronarcys dorsata* (Plecoptera) and *Brachycentrus americanus* (Trichoptera): importance of exposure duration. Environ. Pollut. Series A Ecol. Biol. 35, 353–365. 10.1016/0143-1471(84)90080-1

[B51] Rius S. P. Casati P. Iglesias A. A. Gomezcasati D. F. (2008). Characterization of Arabidopsis lines deficient in GAPC-1, a cytosolic NAD-dependent glyceraldehyde-3-phosphate dehydrogenase. Plant Physiol. 148, 1655–1667. 10.1104/pp.108.128769 18820081 PMC2577239

[B52] Rudneva I. I. (1999). Antioxidant system of black sea animals in early development. Comp. Biochem. Physiol. C. 122, 265–271. 10.1016/S0742-8413(98)10121-4 10190054

[B53] Rukke N. A. (2002). Effects of low calcium concentrations on two common freshwater crustaceans, *Gammarus lacustris* and *Astacus astacus*. Funct. Ecol. 16, 357–366. 10.1046/j.1365-2435.2002.00637.x

[B54] Schägger H. Pfeiffer K. (2001). The ratio of oxidative phosphorylation complexes I-V in bovine heart mitochondria and the composition of respiratory chain supercomplexes. J. Biol. Chem. 276, 37861–37867. 10.1074/jbc.M106474200 11483615

[B55] Schnell J. D. Hicke L. (2003). Non-traditional functions of ubiquitin and ubiquitin-binding proteins. J. Biol. Chem. 278, 35857–35860. 10.1074/jbc.R300018200 12860974

[B56] Semenza G. L. (2004). Hydroxylation of HIF-1: oxygen sensing at the molecular level. Physiology 19, 176–182. 10.1152/physiol.00001.2004 15304631

[B57] Sheehan D. Meade G. Foley V. M. Dowd C. A. (2001). Structure, function and evolution of glutathione transferases: implications for classification of non-mammalian members of an ancient enzyme superfamily. Biochem. J. 360(Pt 1), 1–16. 10.1042/bj3600001 11695986 PMC1222196

[B58] Shen B. Zhang Z. Wang Y. Wang G. Chen Y. Lin P.. (2008). Differential expression of ubiquitin-conjugating enzyme E2r in the developing ovary and testis of penaeid shrimp *Marsupenaeus japonicus*. Mol. Biol. Rep. 36, 1149–1157. 10.1007/s11033-008-9291-7 18581257

[B59] Sloane B. F. (1990). Cathepsin B and cystatins: evidence for a role in cancer progression. Semin. Cancer Biol. 1, 137–152. 2103490

[B60] Song L. Bian C. Luo Y. Wang L. You X. Li J. (2016). Draft genome of the Chinese mitten crab, *Eriocheir sinensis*. GigaScience 5:5. 10.1186/s13742-016-0112-y 26823974 PMC4730596

[B61] Stapleton D. Mitchelhill K. I. Gao G. Widmer J. Michell B. J. The T.. (1996). Mammalian AMP-activated protein kinase subfamily. J. Biol. Chem. 271, 611–614. 10.1074/jbc.271.2.611 8557660

[B62] Sun G. Tarasov A. I. McGinty J. McDonald A. da Silva Xavier G. Gorman T.. (2010). Ablation of AMP-activated protein kinase alpha1 and alpha2 from mouse pancreatic beta cells and RIP2.Cre neurons suppresses insulin release *in vivo*. Diabetologia 53, 924–936. 10.1007/s00125-010-1692-1 20221584 PMC4306708

[B63] Tan M. G. Ooi L. L. Aw S. E. Hui K. M. (2004). Cloning and identification of hepatocellular carcinoma down-regulated mitochondrial carrier protein, a novel liver-specific uncoupling protein. J. Biol. Chem. 279, 45235–45244. 10.1074/jbc.M403683200 15322095

[B64] Tarze A. Deniaud A. Le Bras M. Maillier E. Molle D. Larochette N.. (2007). GAPDH, a novel regulator of the pro-apoptotic mitochondrial membrane permeabilization. Oncogene 26, 2606–2620. 10.1038/sj.onc.1210074 17072346

[B65] Tokeson J. P. E. Holmes J. C. (1982). The effects of temperature and oxygen on the development of *Polymorphus marilis* (Acanthocephala) in *Gammarus lacustris* (Amphipoda). J. Parasitol. 68, 112–119. 10.2307/3281332

[B66] Tong B. Wan B. Wei Z. Wang T. Zhao P. Dou Y.. (2014). Role of cathepsin B in regulating migration and invasion of fibroblast-like synoviocytes into inflamed tissue from patients with rheumatoid arthritis. Clin. Exp. Immunol. 177, 586–597. 10.1111/cei.12357 24749816 PMC4137842

[B67] Truebano M. Tills O. Spicer J. I. (2016). Embryonic transcriptome of the brackishwater amphipod *Gammarus chevreuxi*. Mar. Genomics 28, 5–6. 10.1016/j.margen.2016.02.002 26896099

[B68] Udomsinprasert R. Pongjaroenkit S. Wongsantichon J. Oakley A. J. Prapanthadara L. A. Wilce M. C.. (2005). Identification, characterization and structure of a new delta class glutathione transferase isoenzyme. Biochem. J. 388(Pt 3), 763–771. 10.1042/BJ20042015 15717864 PMC1183455

[B69] Vutukuru S. S. Chintada S. Madhavi K. R. Rao J. V. Anjaneyulu Y. (2006). Acute effects of copper on superoxide dismutase, catalase and lipid peroxidation in the freshwater teleost fish, *Esomus danricus*. Fish Physiol. Bioche. 32, 221–229. 10.1007/s10695-006-9004-x

[B70] Wang G. D. Fan R. X. Zhai W. Liu F. Wang L. Zhong L. (2014). Genetic convergence in the adaptation of dogs and humans to the high-altitude environment of the Tibetan Plateau. Genome. Biol. Evol. 6:2122. 10.1093/gbe/evu162 25091388 PMC4231634

[B71] Wang Y. P. Lu Y. Zhang Y. Ning Z. M. Li Y. (2015). The draft genome of the grass carp (*Ctenopharyngodon idellus*) provides insights into its evolution and vegetarian adaptation. Nat. Genet. 47:962 10.1038/ng0815-962a 26220136

[B72] Wilhelm F. M. Schindler D. W. (2001). Reproductive strategies of *Gammarus lacustris* (Crustacea: Amphipoda) along an elevation gradient. Funct. Ecol. 14, 413–422. 10.1046/j.1365-2435.2000.00426.x

[B73] Winder W. W. Hardie D. G. (1999). AMP-activated protein kinase, a metabolic master switch: possible roles in type 2 diabetes. Am. J. Physiol. 277, 1–10. 10.1152/ajpendo.1999.277.1.E1 10409121

[B74] Wolff C. Gerberding M. (2015). Crustacea: comparative aspects of early development, in Evolutionary Developmental Biology of Invertebrates, ed Wanninger A (Vienna: Springer), 39–61.

[B75] Xi Q. K. Zhang Y. Liu X. Y. Pan P. Sun D. J. (2015). Contents of soluble proteins, and sex hormones, and indicators related to immune during embryonic development of Amur sturgeon *Acipenser schrenckii. J*. *Dalian Fish*. Uni. 30, 357–362.

[B76] Xu P. Zhang X. Wang X. Li J. Liu G. Kuang Y. (2014). Genome sequence and genetic diversity of the common carp, *Cyprinus carpio*. Nat. Genet. 46, 1212–1219. 10.1038/ng.3098 25240282

[B77] Yang W. E. Ho C. C. Yang S. F. Lin S. H. Yeh K. T. Lin C. W.. (2016). Cathepsin B expression and the correlation with clinical aspects of oral squamous cell carcinoma. PLoS ONE 11:e0152165. 10.1371/journal.pone.0152165 27031837 PMC4816521

[B78] Young J. W. (1977). Gluconeogenesis in cattle: significance and methodology. J. Dairy Sci. 60, 1–5. 10.3168/jds.S0022-0302(77)83821-6 320235

[B79] Zadereev E. S. Tolomeyev A. P. Drobotov A. V. Emeliyanova A. Yu Gubanov M. V. (2010). The vertical distribution and abundance of *Gammarus lacustris* in the pelagic zone of the meromictic lakes Shira and Shunet (Khakassia, Russia). Aquat. Ecol. 44, 531–539. 10.1007/s10452-010-9329-5

[B80] Zhang E. Brewer J. M. Minor W. Carreira L. A. Lebioda L. (1997). Mechanism of enolase: the crystal structure of asymmetric dimer enolase-2-phospho-D-glycerate/enolase-phosphoenolpyruvate at 2.0 A resolution. Biochemistry 36, 12526–12534. 10.1021/bi9712450 9376357

